# Valedictory

**Published:** 1851-03

**Authors:** Edwin G. Meek


### Part 4.—Editorial.

### ARTICLE I.

#### VALEDICTORY.

With this number ceases the editorial connection of the undersigned with the N. W. Medical and Surgical Journal. In parting with those whom he has been so long accustomed to address and from many of whom he has received such numerous tokens of good will and friendship the editor feels that he would be derelict in duty were he to dissolve this connection without signifying his sense of obligation to those of his friends who have favorably regarded his labors in behalf of the profession.

It is not necessary to enter into the history of this Journal under the management of the present editors, further than to observe, that when they undertook to conduct it, it labored under very considerable embarrassment. From that embarrassment the profession generously stepped forward and relieved it; thus placing it in a position to render more effective aid in the cause of medical science. In dissolving his connection with it, the subscriber is deeply gratified in being able to assert that the Journal is more prosperous than at any former period of its existence. How far his own efforts have contributed to bring this about, it does not become the undersigned to say. It is enough for him to know that he has retained the good will and confidence of his excellent and esteemed colleague----for whose friendship he can never be sufficiently grateful---and, as he believes, the respect of a great majority of those for whose benefit his editorial labors have been performed.

Whatever of good will or friendship has been manifested towards the writer, during his editorial career, he takes pleasure in bespeaking a continuance of it to the senior editor, whose labors in behalf of medical science render him worthy the esteem of every true son of Æsculapius; and certainly there is no one who more heartily than the undersigned, wishes him brilliant success in his future editorial career.

Amongst those who are and have been readers of this Journal, the writer numbers many warm and personal friends, whose "old familiar faces," he will in all human probability never be again privileged to see. He begs to assure them that he will carry with him to his new home on the Pacific a most grateful appreciation of their kindness and partiality. And to them as well as to those who without personal acquaintance have manifested a generous interest in our enterprise, the writer in bidding them farewell, desires to reiterate his assurance of respect and esteem, and his sincere wishes for their future prosperity and happiness.

February 15, 1851. EDWIN G. MEEK.

